# Reliability and Validity of a Nepali Language Version of the Caregiver Knowledge of Child Development Inventory: A Mixed Method Study

**DOI:** 10.31729/jnma.8952

**Published:** 2025-04-30

**Authors:** Janaki Giri, Neelam Pradhan, Bhawana Shrestha, Rita Shrestha

**Affiliations:** 1 PadmaKanya Multiple Campus, Bagbazar, Kathmandu, Nepal; 2 Dhulikhel Hospital Kathmandu University Hospital, Research & Development Department, Dhukilhel, Kavre, Nepal

**Keywords:** *acceptable*, *child development*, *CKCDI*, *model*, *version*

## Abstract

**Introduction:**

The term "child development" describes the changes a child goes through from birth to adulthood in terms of their physical, linguistic, cognitive, emotional, and other development. Parents, caretakers, and family all have an important effect on it, especially during the first two years. The study aims to translate and analyze the psychometric properties of the English version of the (Caregiver Knowledge of Child Development Inventory (CKCDI) into the Nepali version (CKCDI-N) for mothers of children under two years old.

**Methods:**

This was a cross-section validation study carried out in Kageshwori Manohara Municipality from April 2024 to August 2024. Mothers with children under two years were randomly selected for the study. In first part tools were translated and cross-cultural adaptation was done by focused group discussion. This was followed by psychometric evaluation where Cronbach's alpha, factor analysis, and confirmatory factor analysis were used.

**Results:**

The content validation indicated the use of a reliable test with a Cronbach alpha of 0.704 for developing skills and 0.705 for developmental stimulation. The original CFA model had poor fit, but the updated model had a fair fit after correlating seven error term pairs. Factor loadings were over 0.50 for every item, and the diagonal values representing AVE square roots were greater than other correlation values.

**Conclusions:**

This reliability and validity of the Nepali Version of CKCDI-N showed that every item displayed as acceptable, and the internal consistency was also satisfactory.

## INTRODUCTION

Child development involves physical, linguistic, cognitive, emotional, and other changes from birth to adulthood. Early years are crucial for survival, influenced by parents, caregivers, and families. Child development progresses from dependency, with maternal responsiveness providing predictive validity in developmental science.^1,2,3^

Mothers play a crucial role in children's development, providing stimulation and care, but understanding of child development is often lacking in developing nations.^4,5^ Nepalese moms’ lacks awareness about child development.^6,7^ In Nepal, parents are primary caregivers, particularly mothers, with fathers primarily involved in economic generation. House-making tasks are primarily performed by females.

Caregivers, mostly women, assist with daily tasks like dressing, bathing, medication, and feeding. Measures of internal consistency, intra-rater agreement, and inter-rater agreement classify scale reliability.^8^ Validating the Nepali language version of the CKCDI (Caregiver Knowledge of Child Development Inventory) is necessary for its meaningful application in the Nepali context, where cultural differences and language complexities play key roles in caregiving practices. The study aims to validate the Caregiver Knowledge of Child Development Inventory (CKCDI) in Nepali language.

## METHODS

A cross-sectional validation study was adopted. This descriptive study design was quick and allowed the study to collect data at once, which helped save time for any research. Kageshwori Manohara Municipality was purposively selected. There are nine wards with a male population of 30,021 and a female population of 30,226. The literacy rate was 80.9%. It covers 27.38 sq. km. The most spoken language in the municipality was Nepali.^9^ Three wards were randomly chosen from the total number of wards using the lottery method.

The study duration was six months. The research was conducted with adherence to ethical principles. Authorization was obtained from the study's initial author. Before beginning the data collection process, ethical approval was acquired from the Nepal Health Research Council (Reference number: 1753). Authorities from Kageshwori Manohara Municipality granted permission to conduct the study, and informed consent was obtained for study approval. Each respondent provided written and informed consent before being included in the research project. The data were accessed only by the researcher and were stored confidentially. Before data collection, the study's aim was explained to the participants, and their written consent was obtained. Data obtained from the research sample were kept private, ensuring confidentiality.

The mother's group with children under two years old and mother between 15 to 49 years old^10^ and medical facilities that provided immunization services to children under two years old were included in the study. Three focus group discussions (FGD) were conducted in Kageshwori municipalities. A purposive sampling method was used. Three FDG with representation from the diverse ethnic groups (Brahmin, Chettri, Janajati, Madheshi and Mongolians) was ensured for cross cultural validation. To justify the sample size for psychometric evaluation, a subject-to-item ratio ranging from 5 to 20 was used.^11^

The CKCDI is a comprehensive and user-friendly inventory that was created in 2007 in the Department of Developmental Pediatrics at Ankara University to gauge caregivers’ understanding of infant and early childhood development it has 20 items and takes about five minutes to complete. The "International Guide for Monitoring Child Development (GMCD)" was used to calculate the age range for each item on the scale. If a caregiver's responses are within the age range for each item, they obtain a score of "2"; if not, they receive a score of "1," which means that the responses are one month out of date. The remaining answers were all determined to be incorrect and were given a score of "0”. The higher the outcome score, which goes from 0 to 40, the more knowledge the caregiver has (Supplementary 1).^12^

The translation procedure was followed from the conducted in Guidelines for translating and adapting psychological instruments.^13^ The adapted tool was first translated from the primary language (English) to the target language (Nepali) by two independent translators fluent in both languages. In the second step, the initial translation was reviewed and finalized through 3 group discussion) in which each item was thoroughly and equally discussed for clarity, cultural adaptation, translation, and comprehensibility.^14^

For content validation clinical psychologists were invited to participate in the study. A minimum of five years of professional experience with a Bilingual translator was included. Clinical psychologists were requested to rate each item according to its clarity, comprehensibility, interpretation, and language performed back translations from the target language into the primary language. A third expert, proficient in both the primary and target languages but blind to the original version, compared the target and primary language translations. The lecturer proofread the material before completing the final CKCDI-N version and the things were finalized with the guidance of a supervisor. The final step in obtaining the Nepali version of the CKCDI was to revise and finalize the target language.

Following the translation procedure (Supplementary 2) the psychometric evaluation was done for which the validated questions were administered to mother's group. Epi Data version 3.1 was utilized for data entry.^15^ Data was coded, examined, and evaluated according to the study's objectives. The completeness, consistency, and accuracy of all the data gathered were examined, checked, and confirmed every day and the socio demographic features were reported using descriptive statistics. The age range for each item on the scale was computed using the "International Guide for Monitoring Child Development (GMCD)”. Are givers receive a score of "2" if their answers fall within the specified age range for each item; otherwise, they receive a score of "1," indicating that the answers are one month outdated. Every other response received a score of "0" and was determined to be inaccurate. The result scores range from 0 to 40 and the higher the score, the more information the caregiver possesses.^12^

Item reliability was also examinedusing the content validity index (I-CVI) and average scale content validity index (S-CVI/Ave) to calculate Cronbach's alpha (CA) and composite reliability (CR). Four clinical psychologists with more than five years of experience dealing with children shared expertise and input. Trial version 29.0 of the IBM Statistical Package for Social Sciences.^16^ The free trial version of IBM SPSS Amos.^17^ was utilized to assess discriminant, convergent and confirmatory factor validity. Microsoft Office Excel 10 was used to calculate the socio demographic variables. The findings are displayed in tabular form.

Cronbach alpha was calculated using the internal consistency of the CKCDI-N. The Cronbach's alpha value was considered moderate and acceptable if it fell between 0.60 and 0.80, alpha values were described as excellent (0.93-0.94), not satisfactory (0.4-0.55) and low (0.11).^20^ The CKCDI-N content validity was assessed by calculating the average Scale Content Validity Index which included Scale Content Validity Index - Average(S-CVI/Ave), Scale Content Validity Index - Universal Agreement (S-CVI/UA), and the Item Content Validity Index (I-CVI). Content validity scale for S-CVI/UA and S-CVI/Ave of 0.8 and 0.9, respectively, and I-CVIs of 0.78 or higher was considered ideal.^19^

The Root Mean Square Error of Approximation (RMSEA) is used to assess model fit in factor analysis. RMSEA values less than 0.05 indicate a close fit, values between 0.05 and 0.08 suggest a fair fit, and values higher than 0.10 are considered to indicate a poor fit. The model fit acceptance criteria include a chi-square to degrees of freedom ratio (X^2^/DF) of less than 3, a p-value less than 0.000, a Comparative Fit Index (CFI) of 0.9 or higher, a Goodness-of-Fit Index (GFI) of at least 0.92, an RMSEA of 0.08 or lower, and a Root Mean Square Residual (RMR) of 0.05.^18^ To evaluate construct validity, factor analysis was conducted following these criteria.

The variables were determined using percentages and frequency of socio demographic variables. For Convergent validity a factor loading over the 0.50 threshold.^20^ suggested was computed. For establishing discriminant validity the Fornell and Larcker (1981) criteria was used. The diagonal values are bold, which is square root of AVE (average variance extraction ) and the other values are inter-variable correlation. The Fornell-Larcker criterion states that each latent variable's square root of AVE must be greater than any other correlation value among the latent variables.^21,22^

## RESULTS

In first FGD, 8 mothers from the ethnic background Brahmin and Chettri participated their age was between 15-35 years; in second FDG 6 mothers from janajati ethnic back ground participated, their age was between 25-35 years. Similarly, in third FDG six mothers with ethnic background Madhesi and Mangolian participated, their age was between 30-42 years. The obstacle faced during the focus group discussion was the location because the discussion was conducted in maternal room, sometimes the maternal regular check has to do. Engagement of mothers with their children interrupted and disturbs the setting. Although the disturbances occurred the focus group discussion was conducted tactfully and results successful.

Focus group discussion 1: Most of the mothers had knowledge about the physical, linguistic and emotional changes occurs of child but were not aware about the age range of the child development. And, the questions were asked about their children relating their activities *(dimakha ko biskas, akha ghumayera herne, artha lagne sabdha bolna* ), they showed an interest awareness of the concept. Majority of the respondents never practice *(chusaune kura)* but they were familiar about the practice. Probing about not practicing *(khai ta hunna khai faida xaina khana payee ko hunu)* no nutritional value. Spoon or a fork are not given to the child because the child might get hurt. We prefer to give child to eat food by hands rather using spoon or a fork said by the respondents.

Focus group discussion 2: In focus group discussion, participants were different education background (science stream and arts). The level of education was from secondary to the master. Mothers had knowledge about the developmental process occurs after the birth of child. They were more concern about the physical growth of the child. However one of the respondent never give spoon because that might cause injury to the child *(Akha ghochxani).*

Focus group discussion 3: Mothers group were knowledge about the child's cognitive and social- emotional development (*dimakhako bikashunxa, bolna sikchan yo belama*). Mothers group don't give their child a spoon or a fork to let them eat by themselves *(chamchaa kata ta dinnau baru hatale nai khana sikaune gareka xau, haat ni vaanu parena aafai khana dina vane vaihalxa ni sajilo ni cha ).* In discussion, mothers group suggested to remove (*Chamcha prayog*) from the sentence. Most of the participants said that we don't practice giving spoon and fork to the children. Participants no 1 &3,4 from FGD1 &FGD2 respectively said that "*ghau hunxa aakha gojcha".* All three groups agreed to remove the word from the sentence because the eating practice is given with hand rather than spoon. The word is not suitable for the cultural context said by the participants.

The majority of mothers understood physical, linguistic, and emotional development but was not aware of the precise age ranges for developmental milestones. They demonstrated interest when discussing their children's activities (e.g., cognitive growth, eye-tracking, and early speech). All focus group discussions involved mothers of children less than two years. Following focus group discussions, some items were changed to better reflect mothers’ experiences and practices based on their input.

The study included 625 with children under three years old as subjects for psychometric evaluation. Of the participants, 22 chose not to participate, 28 did not finish the questionnaire and 15 were removed from the study due to important missing data like the mother's age, the child's age, her employment status, and her level of education. 560 samples had been thoroughly examined and statistically assessed.

**Table 1:** Demographic characteristics of mother participating in psychometric assessment of Caregiver Knowledge of Child Development Inventory (n=560).

Age of the mother (years)	N (%)
15-19	30 (5.36)
20-24	155 (27.68)
25-29	224 (40.00)
30-34	119 (21.25)
35-39	28 (5.00)
40-44	4 (0.71)
**Age of child (months)**	
< 6	203 (36.25)
6-11	225 (40.18)
12-23	118 (21.07)
< 24	14 (2.50)
**Gender of child**	
Female	283 (50.54)
Male	277 (49.46)
**Number of children in family**	
First	333 (59.46)
Second	203 (36.25)
Third or more	24 (4.29)
**Maternal education**	
No Literate	3 (0.54)
Primary (1-5)	7 (1.25)
Upper primary (6-8)	19 (3.39)
Secondary (9-10)	71 (12.68)
Higher secondary (11-12)	312 (55.71)
Bachelor	74 (13.21)
Master	74 (13.21)
**Maternal employment**	
House maker	405 (72.32)
Service	75 (13.39)
Business	80 (14.29)

The sample was evenly divided between male and female, with 277 (49.5%) male and (50.3%) female children (Table 2) The Cronbach alpha was 0.704 for developmental skills and 0.705 for developmental stimulation (Table 2).

**Table 2:** Internal Consistency of CKCDI-N

Constructs	Items	Cronbach Alpha	Composite Reliability
Developmental Skills	N(1-10)	0.704	0.705
Developmental Stimulation	N(11-20)	0.701	0.702

Reliability test, as indicated by the content validation, or I-CVI and SCVI/Ave, were both 1 (Table 3).

The Confirmatory Factor Analysis (CFA) results indicated that the model did not achieve a good fit, as some fit indices did not meet the acceptance criteria. The model fit results were X^2^ = 601.641, DF = 169, X^2^/DF = 3.56, p < 0.000, CFI = 0.74, GFI = 0.92, RMSEA = 0.068, and RMR = 0.043. Based on the predefined model fit criteria (x^2^/DF < 3, p < 0.000, CFI ≥ 0.9, GFI ≥ 0.92, RMSEA ≤ 0.08, and RMR = 0.05), the Goodness-of-Fit Index (GFI = 0.92), Root Mean Square Error of Approximation (RMSEA = 0.068), and Root Mean Square Residual (RMR = 0.043) met the threshold levels, indicating an acceptable model fit in these aspects. However, Chi-square/DF (3.56) exceeded the recommended limit of <3, and the Comparative Fit Index (CFI = 0.74) was below the acceptable threshold (≥0.9), suggesting an overall lack of model fit.

To improve the model fit, covariances were drawn between specific item pairs (N2 with N1, N3 with N2, N5 with N4, N8 with N5, N18 with N17, N19 with N16, and N20 with N11). This approach enhances Confirmatory Factor Analysis (CFA) and Structural Equation Modeling (SEM) by accounting for shared measurement errors across related components, thereby increasing the model's accuracy (Table 4), (Supplementary 3).

For each item, the factor loading was greater than 0.50 in each item ranging from 0.7 to 1.3. Item numbers N1, N19, and N20 had factor loadings exceeding the standard threshold. Statistically significant loadings above the 0.50 cutoff indicate that these items contribute meaningfully to the constructs. Except for N1, N19, and N20, all factor loadings met the required criteria. Items were not removed to maintain interpretability and scoring structure due to potential model-specific estimation issues, rather than actual measurement error (Table 5).

Among the latent variables, the diagonal values (0.69 and 0.71) corresponding to the square roots of AVE were larger than the other correlation values (0.24 and 0.24).

**Table 3:** Internal Consistency of CKCDI-N.

Items	Expert 1	Expert 2	Expert 3	Expert 4	Expert in agreement	I-CVI	SCVI/Ave
N1	1	1	1	1	4	1	1
N2	1	1	1	1	4	1	1
N3	1	1	1	1	4	1	1
N4	1	1	1	1	4	1	1
N5	1	1	1	1	4	1	1
N6	1	1	1	1	4	1	1
N7	1	1	1	1	4	1	1
N8	1	1	1	1	4	1	1
N9	1	1	1	1	4	1	1
N10	1	1	1	1	4	1	1
N11	1	1	1	1	4	1	1
N12	1	1	1	1	4	1	1
N13	1	1	1	1	4	1	1
N14	1	1	1	1	4	1	1
N15	1	1	1	1	4	1	1
N16	1	1	1	1	4	1	1
N17	1	1	1	1	4	1	1
N18	1	1	1	1	4	1	1
N19	1	1	1	1	4	1	1
N20	1	1	1	1	4	1	1
Proportion	1	1	1	1	1		
Relevance	1	1	1	1			

**Table 4:** Model fitness initial and final.

	Initial model			Final model	
Measures	Threshold	Result	Threshold	Result	Acceptable level^18^
CHI-SQUARE	3.56	Not achieved	2.84	Achieved	<3
GFI	0.92	Achieved	0.95	Achieved	>0.92
RMR	0.04	Achieved	0.034	Achieved	<0.05
RMSEA	0.06	Achieved	0.057	Achieved	<0.08
CFI	0.74	Not achieved	0.909	Achieved	>0.9

**Table 5:** Final model fit.

Constructs	Standard Factor Loadings	Average Variance Extracted (AVE)	Square Root of Average Variance Extracted
Developmental Skills		0.383	0.619
N1	1		
N2	0.7		
N3	0.8		
N4	0.9		
N5	1		
N8	1		
Developmental Stimulation		0.51	0.71
N11	1		
N15	0.8		
N16	0.8		
N17	1		
N18	0.9		
N19	1.3		
N20	1.2		

## DISCUSSION

The study was conducted between April 2024 to August 2024 with the aim to translate and analyze the psychometric properties of the English version of the CKCDI into the Nepali version (CKCDI-N) for mothers of children under two years old. The study aimed to translate and analyze the psychometric properties of the English version of the CKCDI into the Nepali version (CKCDI-N) for mothers of children under two years old. Using observation, interviews, and archival records, the Nepali version was developed through multiple trials in 560 samples.

A small study with 30 participants tested research protocols, data collection instruments, and recruitment strategies. For larger studies, 10% of the required sample size and 30-50 participants are recommended, or more than survey questions.^25^ In the pilot study, most of the participants were 20-40 years’ age group mothers. The internal consistency of the overall 20-item scale was 0.61.^12^ The CA value was considered moderate and acceptable if it fell between 0.6 and 0.80.^26^ CA for developmental skills 0.6 and developmental stimulation 0.61 Hence, constructs are acceptable for the final test. The study recommended that a scale with excellent content validity should be composed of I-CVIs of 0.78 or higher and S-CVI/UA and S-CVI/Ave of 0.8 and 0.9 or higher, respectively. ^27^ The content validation i.e. I-CVI is 1, SCVI/Ave is 1 which indicates that used inventory is an acceptable reliability test. Alpha values were described as excellent (0.93-0.94), not satisfactory (0.4-0.55) and low (0.11).^25^

In the final study The CA was 0.704 for developmental skills and 0.705 for developmental stimulation which was acceptable for inventory. In this study, the total mean score was 16.92 but the mean CKCDI questionnaire score was 19.2. ^28^ Since, the total mean score is lower than the original article.^12^

Internal consistency refers to how closely all of the test's items assess the same idea or construct, and it is therefore related to how closely the test's items are related to one another. ^29^ The CA reliability for developmental skills and developmental stimuli (0.74 and 0.71) respectively was found in our study. For all the measurements, the CA was acceptable. ^30^ CR is a superior internal consistency that prevents underestimating, which is a common problem with CA. A CR of 0.6 is appropriate.^31^ CR for developmental skills was found 0.705 and developmental stimulation was 0.702 hence, it is appropriate construct.

Using a distinct set of criteria to determine whether the construct of interest is suitable, CFA is a statistical method of testing the internal structure of instruments.^29^ The model is used for testing the reliability, convergent validity, and discriminant validity. The recommended values are based on the guidelines. ^18^ The CFA indicated that the model was less than a good fit. (X^2^ = 601.641, DF. = 169, X^2^/DF = 3.56, p<.000, CFI =0.74, GFI =0.92, RMSEA =0.068, RMR=0.043 ).^21^ The measures (GFI,RMR and RMSEA) were within the threshold level whereas Chi-square and CFI results were not achieved. Therefore, when the modification was made, several pairs of error terms were found when modification indices were examined; if correlation were to considerably lower the chi-square value, these terms would be disclosed. If correlations are low and do not alter estimations of the remaining parameters, then correlating error terms is permitted.^32^ There was correlation between 7 pairs. Following the correlation of the error terms, the adjusted two-factor measurement model was found to have a good fit according to all fit indices (X2 = 162.02, DF. =57, p <.000, X2 / DF = 2.8, CFI =0.90, GFI =0.95, RMSEA =0.05, RMR=0.034).^21^

For Convergent validity a factor loading over the 0.50 threshold ^20^ which was computed. In our study, there was more than a 0.50 factor loading for each item. The statistically appropriate range for loading exogenous variables onto latent variables is 0.50 to 0.95.^33^ The loadings of the factor and items range from 0.7 to 1.3, suggesting small error. Item number N1, N19, N20 consists greater than 1 standard factor loading. The items are maintained due to multicollinearity in scoring structure and interpretability. The 2-dimensional model performs best in confirmatory factor analysis. Current research focuses on determining multicollinearity origin, severity, managing high-dimensional data, and addressing nonlinear interactions. ^34^ Researchers are still exploring the most effective approaches to deal with multicollinearity in factor analysis and are striving to develop more comprehensive techniques.^35^

For establishing discriminant validity.^36^ Fornell-Larcker criterion was used. The diagonal values are bold, which is square root of AVE and the other values are inter-variable correlation. The Fornell-Larcker criterion states that each latent variable's square root of AVE must be greater than any other correlation value among the latent variables.^30^ The diagonal values (0.69 and 0.71) which are square roots of AVE are greater than the other correlation values (0.24 and 0.24) among the latent variables. Thus, variables have discriminant validity.

Despite its strengths, the study has certain limitations. The CKCDI-N primarily assesses maternal knowledge and may not fully capture external factors influencing child development. Additionally, reliance on selfreported data may introduce bias, and the crosssectional nature of the study limits the ability to establish causal relationships. Future research should explore longitudinal assessments.

## CONCLUSIONS

This reliability and validity of the Nepali Version of CKCDI-N showed that every item displayed acceptable, and the internal consistency was also satisfactory.
